# D6 high-quality expanded blastocysts and D5 expanded blastocysts have similar pregnancy and perinatal outcomes following single frozen blastocyst transfer

**DOI:** 10.3389/fendo.2023.1216910

**Published:** 2023-11-09

**Authors:** Juwei Hu, Juan Zheng, Jie Li, Haiyue Shi, Hua Wang, Bangxu Zheng, Kun Liang, Chunhao Rong, Liming Zhou

**Affiliations:** Reproductive Medicine Center, Ningbo Women and Children’s Hospital, Ningbo, Zhejiang, China

**Keywords:** frozen-thawed blastocyst transfer, pregnancy outcomes, perinatal outcomes, day 5 versus day 6, blastocyst vitrification

## Abstract

**Objective:**

We compared the pregnancy and perinatal outcomes between expanded blastocysts vitrified on D5 versus D6 following single frozen blastocyst transfer.

**Methods:**

Clinical data on 7,606 cycles of frozen-thawed blastocyst implantations were retrospectively analyzed. Depending on whether blastocysts were vitrified on D5 or D6 and the transferred blastocysts, the blastocysts were divided into 6 groups: HQB-D5, HQB-D6, 4XC-D5, 4XC-D6, 4CX-D5, and 4CX-D6 groups. The differences in clinical pregnancy rate, live birth rate, first trimester abortion rate, preterm birth rate, gestational age, birth weight, and sex ratio at birth among the groups were compared.

**Results:**

Our study showed that there was no difference in pregnancy and perinatal outcomes between the delayed formation of D6 high-quality expanded blastocysts and D5 expanded blastocysts, whether they were high-quality blastocysts or not. For low-quality blastocysts, the clinical pregnancy rate of D5 was higher than that of D6, and D5 was also better than D6 in live birth rate for those with inner cell mass rating B or above, while there was no difference between D5 and D6 for those with inner cell mass rating C.

**Conclusion:**

Based on our research, we suggest that when we are developing the implantation strategy, we give priority to the selection of high-quality expanded blastocysts, regardless of D5 and D6, whose clinical outcomes are not different. For low-quality blastocysts, D5 expanded blastocysts are preferred for transfer.

## 1 Introduction

In recent years, with the development of assisted reproductive technology, the *in vitro* culture system has been optimized and upgraded, and the blastocyst formation rate has been greatly improved. The implantation strategy has been gradually transformed from the traditional cleavage stage to the blastocyst stage. This transformation led to single blastocyst implantation, which not only improves the clinical pregnancy rate but also provides a new strategy for reducing multiple pregnancy rate. Single blastocyst transfer can ensure a higher clinical pregnancy rate and a lower multiple pregnancy rate than traditional embryo transfer at the cleavage stage (1, 2). The development and implementation of vitrification have broken the rules of *in vitro* fertilization (IVF) technology. Vitrification directly contributed to wide-spread acceptance of elective single embryo transfer, which has led to a sharp decline in the rates of twins and higher order multiple gestations from IVF treatment (3). In particular, the number of reproductive centers selecting a single blastocyst transfer during the frozen-thawed transfer cycle has been increasing.

In general, on D5 after oocyte retrieval, the cleavage-stage embryo develops into a blastocyst. Because of a difference in the rate of embryo development, some embryos form a blastocyst on D6 after oocyte retrieval. D5 refers to approximately 120 hours after insemination, and D6 is approximately 144 hours. These blastocysts have high developmental potential and planting capacity and show an increased likelihood of a successful pregnancy. However, whether delayed blastulation affects the developmental potential of the embryo is not known. Moreover, the effects of the day of the blastocyst formation and vitrification on pregnancy and perinatal outcomes are still controversial, and the current literature shows inconsistent results.

A systematic review and meta-analysis showed that, slower developing blastocysts cryopreserved on D6 but at the same stage of development as those developing to the blastocyst stage on D5 have similar clinical pregnancy rate, ongoing pregnancy rate and live birth rate (LBR) following frozen-thawed blastocyst transfers (4). While Alice Tubbing et al. demonstrated a significant difference in clinical pregnancy (43% versus 23%, P < 0.001) and LBR (34% versus 16%, P < 0.001) regarding the day of vitrification, in favor of day 5 after adjustment for confounding factors, when the stage of development of the blastocyst was taken into consideration, they still observed a significant advantage of D5 versus D6 vitrification (5). Another meta-analysis showed that, for blastocyst-stage embryo transfer, the pregnancy rate and implantation rate were both higher formed by D5 than those formed by D6, the superiority of D5 blastocyst-stage embryo transfer can be influenced by the chromosomal status of embryos (6, 7). On the other hand, Yang H et al. and EI-Toukhy T et al. demonstrated that high-quality D6 blastocysts in frozen-thawed cycles had similar developmental potential and pregnancy outcomes compared to those of high-quality D5 blastocysts (8, 9). Kaye et al. suggested that single D6 blastocyst transfers have similar clinical pregnancy rates and ongoing pregnancy rates as D5, especially after vitrification (10).

At present, in our study, expanded blastocysts at the same stage (stage 4) were selected as the research objects. Early blastocysts or blastocysts stage 3/5/6 were excluded, as the effect of blastocyst expansion on the LBR is also controversial, and the speed of embryo development determines that the majority of blastocysts are stage 4 blastocysts on D5 or D6. In our center, most blastocysts were transferred at stage 4, and the number of transferred blastocysts at other stages was small enough to be ignored, so we took stage 4 expanded blastocysts as our study subjects. Systematic comparisons were made according to blastocyst quality, grade of ICM (inner cell mass), and the day of vitrification in the frozen-warmed blastocyst transfer cycle. The findings of this study are expected to further reveal the influence factors of embryo development potential, facilitate the selection of embryos in the frozen-thawed blastocyst transfer cycle for doctors and patients.

## 2 Methods

### 2.1 Patient cohort and study design

**Figure 1:**
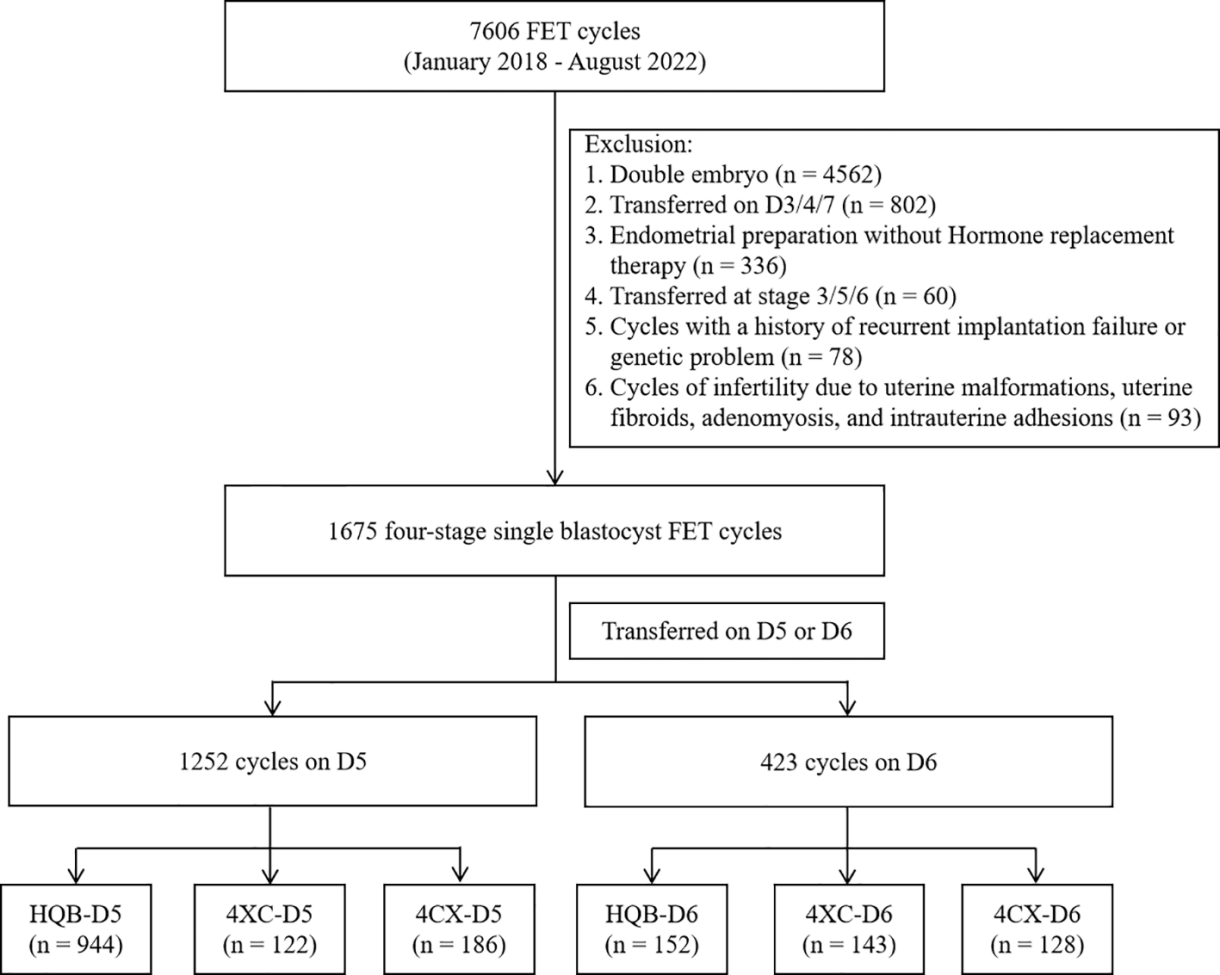
Flow chart showing the data selection process for analysis in the study.

In total, 7,606 cycles of frozen-thawed embryo transfer were performed at our center between January 2018 and August 2022. The inclusion criteria in this study were as follows: (i) FET (frozen embryo transfer) cycle implantation of a single blastocyst; (ii) blastocysts cultured to D5 or D6 *in vitro* and graded at stage 4 on morphology; (iii) absence of a history of genetic conditions in both men and women, (iv) Hormone replacement therapy was used to prepare the endometrial for women undergoing FET. The exclusion criteria were as follows: presence of (ii) a history of repeated implantation failure; (i) a history of habitual abortion; and (iii) a history of uterine malformation, hysteromyoma, adenomyosis and intrauterine adhesion. Transfer cycles in which double embryos were transferred (n = 4,562), were transferred on D3/4/7 (n = 802), were replaced in a natural cycle without artificial endometrial programming (n = 336), were transferred at stage 3/5/6 (n = 60), or had a history of recurrent implantation failure or a genetic problem (n = 78) were excluded. Cycles of infertility due to uterine malformations, uterine fibroids, adenomyosis, and intrauterine adhesions (n = 93) were also excluded. Finally, 1,675 cycles were included in the study (**Figure 1**). This study was approved by the ethics committee of Ningbo Women and Children’s Hospital (ID: EC2022-M013).

The expanded blastocysts included in the study were divided into 6 groups according to the implanted blastocysts and the day of blastocyst vitrification: groups of implanted blastocysts (4BB or above) on D5 (HQB-D5), 4BB or above on D6 (HQB-D6), 4AC/4BC on D5 (4XC-D5), 4AC/4BC on D6 (4XC-D6), 4CA/4CB on D5 (4CX-D5), and 4CA/4CB on D6 (4CX-D6).

### 2.2 Blastocysts culture and scoring

Blastocyst culture was conducted by the sequential culture method. D3 cleavage embryos were transferred to Sydney IVF Blastocyst Medium (COOK) for blastocyst culture. Blastocyst formation was observed on D5 and D6. Blastocyst scoring was performed according to the Gardner scoring system, which takes into account the expansion grade as well as the development of the ICM and the trophectoderm (11). In our study, high-quality blastocysts (HQB) were defined as blastocysts grading AA/AB or BA/BB and expansion grade 4, low-quality blastocysts (LQB) were defined as blastocysts grading AC/BC or CA/CB and expansion grade 4.

### 2.3 Blastocysts vitrification and thawing procedures

Our center’s blastocysts vitrification policy is to proceed on D5 when the ICM and trophoblast cells are clearly visible in a 40×eyepiece. If not, culture is continued until D6, then cryopreservation is performed if the embryo achieves sufficient development and quality. Ten minutes before freezing, a single 1.2 ms laser pulse is applied between two trophoblast cells as far away as possible from the ICM.

The blastocysts were frozen and thawed using KITAZATO Corporation’s freezing and thawing kits (VT101, VT102). Vitrification freezing and thawing operations on blastocysts were carried out in accordance with the operation instructions. Briefly, the blastocysts were incubated in ES solution for 9 minutes and then transferred to VS solution, which was repeatedly cleaned in VS solution until ES solution was completely removed for no more than 60 seconds. Then about 2 µl of blastocyst-wrapped droplets were transferred to the tip of the cryotube and immediately put into liquid nitrogen. The cryotube was then capped in liquid nitrogen and stored in liquid nitrogen.

The cryotube to be thawed was found in the liquid nitrogen, the cap was removed and quickly placed in the 37 ℃ TS solution, which is incubated in the incubator for 30 minutes in advance. After 60 seconds, the blastocysts were transported to DS solution at room temperature for 3 minutes, then to WS1 solution for 5 minutes, and then to WS2 solution for 5 minutes. Finally, the blastocysts were transferred to the Sydney IVF Blastocyst Medium (COOK) solution and placed in an incubator for implantation.

### 2.4 Endometrial preparation

Hormone replacement therapy was used to prepare the endometrium for women undergoing FET. Starting on the second day of menstruation, patients began taking oral estradiol valerate at 6 mg per day for 7 days, followed by 8 mg per day for another 7 days. The endometrium was monitored with B-mode ultrasound after oral estradiol administration. When the endometrium reached a thickness of 7 mm, an intramuscular P injection of 40 mg twice a day was started. If not adequate, endometrial estrogen priming continued, and ultrasound assessment was undertaken to confirm further endometrial thickening.

### 2.5 Blastocysts implantation

The blastocysts were thawed and cultured for about 3 hours before they were implanted with Sydney IVF Blastocyst Medium (COOK) under the guidance of the B-mode ultrasound, and a COOK catheter was used for implantation. After implantation, each patient lay in silence for 60 minutes.

### 2.6 Luteal support program

In patients with hormone replacement therapy cycles, we administered 8 mg E2 valerate daily, 90 mg Crinone (Merck Serono) vaginally once a day. The serum β-human chorionic gonadotropin level was examined 14 days after implantation. Luteal support was maintained until 10 weeks after pregnancy and can be extended if vaginal bleeding is present.

### 2.7 Cycle outcome measures

Patients who were biochemically diagnosed with pregnancy were examined 2 weeks later for intrauterine pregnancy, the presence of embryos and heartbeats in the pregnancy sacs. The presence of pregnancy sacs was considered to indicate clinical pregnancy. LBR was the proportion of newborns whose mothers were ultimately able to successfully deliver a live birth. First trimester abortion rate was the proportion of miscarriages within 12 weeks. Preterm birth rate was defined as the proportion of live births delivered within 37 weeks. Gestational age was defined as the number of pregnancy weeks from the 19th day before FET to delivery. Low birth weight was defined as birth weight less than 2,500 g, and macrosomia was defined as birth weight greater than 4,000 g.

### 2.8 Statistical analysis

The data was statistically analyzed using IBM SPSS 20.0 software (Armonk, NY: IBM Corp.). Quantitative data, which was analyzed by the Kolmogorov-Smirnov test and showed an abnormal distribution, was expressed as the median and interquartile range and tested using the Kruskal-Wallis H(K) test. Comparisons between two groups were compared using the Mann-Whitney U test. Categorical data are expressed as percentages (%), and the Pearson chi-square test was used for comparison between two groups. If the cell theoretical frequency was < 5, an adjusted chi-square test method or likelihood ratio test was adopted. Single factor regression analysis was used to screen the variables that affected LBR, and unadjusted OR (odds ratio) and 95% CI (confidence interval) were obtained. Variables with a P value less than 0.1 were included in the multivariate regression analysis to obtain the adjusted OR and 95% CI. A P-value < 0.05 indicated that a difference was statistically significant among groups; between-group comparisons required comparison with an adjusted P-value P’ (P’ = P/N, N = Number of tests between groups) according to Bonferroni’s adjustment method, so P’ < 0.003 was considered statistically significant for multiple pairwise comparisons.

## 3 Results

### 3.1 General characteristics

A total of 1,675 cycles were included in the study, and the Kruskal-Wallis H (K) test was performed on female age, infertility duration, female body mass index (BMI), anti-mullerian hormone (AMH), basal follicle-stimulating hormone (FSH) level, and endometrial thickness. The results were shown in **Table 1**, statistical results showed no significant differences in female age, infertility duration, female BMI, basal FSH, endometrial thickness, and types of infertility among groups except basal AMH. Highly significant differences in basal AMH among groups were observed (P < 0.001). Analyses between subgroups were performed in basal AMH. The results were showed in **Table 1** and **Supplementary 1**. AMH was significantly different between HQB-D5 and 4XC-D5, 4CX-D5, 4XC-D6, and 4CX-D6. There was no significant difference in any other subgroup.

**Table 1:** Baseline characteristics of female patients.

Parameter	HQB-D5 (n = 944)	HQB-D6 (n = 152)	4XC-D5 (n = 122)	4XC-D6 (n = 143)	4CX-D5 (n = 186)	4CX-D6 (n = 128)	P
Female age (years)	30 (28,33)	30 (28,33)	30 (28,33)	31 (28,34)	31 (28,34)	31 (28,34)	0.082
Infertility duration (years)	3.0 (2.0,5.0)	3.4 (2.2,5.4)	3.5 (2.0,4.5)	3.6 (2.3,5.9)	3.2 (2.0,5.2)	3.5 (2.5,5.3)	0.113
Female BMI (kg/m^2^)	22.0 (20.0,24.0)	21.0 (19.9,23.5)	21.1 (19.5,23.6)	21.8 (20.2,24.1)	21.6 (19.9,23.5)	22.0 (19.5,24.0)	0.294
Basal AMH level (ng/ml)	5.0 (3.0,8.0)	4.4 (2.7,6.5)	3.8 (2.4,6.0)^a^	3.7 (2.0,5.9)^a^	3.9 (2.2,6.0)^a^	3.4 (2.0,5.8)^a^	< 0.001
Basal FSH level (mIU/ml)	7.0 (6.0,8.0)	7.3 (6.1,7.4)	7.3 (6.1,8.0)	7.3 (6.2,8.0)	7.3 (6.2,8.0)	7.3 (5.9,7.9)	0.128
Endometrial thickness (mm)	9.0 (8.0,10.0)	9.0 (8.0,10.0)	9.0 (8.0,10.0)	9.0 (8.0,10.0)	9.0 (8.0,10.0)	9.0 (8.0,10.0)	0.498
Types of infertility							0.827
Primary infertility, n (%)	533 (56.5)	83 (54.6)	73 (59.8)	81 (56.6)	97 (52.2)	70 (54.7)	
Secondary infertility, n (%)	411 (43.5)	69 (45.4)	49 (40.2)	62 (43.4)	89 (47.8)	58 (45.3)	

HQB, high-quality blastocysts; BMI, body mass index; AMH, anti-mullerian hormone; FSH, follicle-stimulating hormone; D5, day 5; D6, day 6. a, compared with HQB-D5, P’ < 0.001. Values are median (1st quartile, 3rd quartile) or n (%).

### 3.2 Pregnancy and perinatal outcomes

Clinical pregnancy rate, LBR, first trimester abortion rate, preterm birth rate, and male/female ratio among groups were compared using the Pearson chi-square test. Birth weight and gestational age among groups were compared using the Kruskal-Wallis H (K) test. Birth defects, low birthweight, and macrosomia were compared using the likelihood ratio test. The results were shown in **Table 2**. Clinical pregnancy rate, LBR and first trimester abortion rate were statistically significant among groups. There were no significant differences in preterm birth rate, gestational age, birth weight, low birthweight, macrosomia, sex ratio, birth defect, or neonatal death among groups.

**Table 2:** Pregnancy and perinatal outcomes on D5 versus D6 in all blastocyst groups.

Parameter	HQB-D5 (n = 944)	HQB-D6 (n = 152)	4XC-D5 (n = 122)	4XC-D6 (n = 143)	4CX-D5 (n = 186)	4CX-D6 (n = 128)	P
Clinical pregnancy rate, n (%)	606 (64.19)	94 (61.84)	70 (57.38)	49 (34.27)^abcd^	111 (59.68)	45 (35.16)^abcd^	< 0.001
LBR, n (%)	515 (54.56)	77 (50.66)	57 (46.72)^e^	31 (21.68)^fg^	90 (48.39)^e^	41 (32.03)^fg^	< 0.001
First trimester abortion rate, n (%)	76 (12.54)^h^	15 (15.96)	11 (15.71)	15 (30.61)	19 (17.12)	4 (8.89)	0.014
Preterm birth rate, n (%)	51 (9.86)	9 (11.69)	6 (10.53)	1 (3.23)	16 (17.78)	5 (12.20)	0.221
Gestational age (wk)	38.2 (38.0,39.1)	38.6 (37.9,39.1)	38.9 (38.1,39.9)	38.4 (37.7,39.0)	38.6 (37.6,39.3)	38.9 (38.1,39.6)	0.105
Birth weight (g)	3400 (3100,3650)	3400 (3050,3700)	3250 (2870,3650)	3400 (3250,3725)	3350 (3000,3550)	3400 (3100,3700)	0.384
Low birthweight, n (%)	17 (3.30)	1 (1.30)	5 (8.77)	2 (6.45)	6 (6.67)	1 (2.44)	0.198^m^
Macrosomia, n (%)	24 (4.66)	4 (5.19)	3 (5.26)	3 (9.68)	5 (5.56)	3 (7.32)	0.890^m^
Male/Female ratio (%)	316/199 (1.59)	48/29 (1.66)	26/31 (0.84)	16/15 (1.07)	49/41 (1.20)	19/22 (0.86)	0.081
Birth defect (n)	7	0	0	1	2	1	0.421^m^
Neonatal death (n)	2	0	0	0	0	0	0.874^m^

HQB, high-quality blastocysts; LBR, live birth rate; D5, day 5; D6, day 6. a, compared with 4XC-D5, P’ < 0.001; b, compared with 4CX-D5, P’ < 0.001; c, compared with HQB-D6, P’ < 0.001; d, compared with HQB-D5, P’ < 0.001; e, compared with 4XC-D6, P’ < 0.001; f, compared with HQB-D6, P’ < 0.001; g, compared with HQB-D5, P’ < 0.001; h, compared with 4XC-D6, P’ < 0.001. m, the result was obtained by the likelihood ratio test. Values are n, n (%) or median (1st quartile, 3rd quartile).

Analyses between subgroups were performed in clinical pregnancy, LBR and first trimester abortion rate. The results were shown in **Table 2** and **Supplementary 2** (P’ < 0.003 was considered statistically significant for multiple pairwise comparisons). The clinical pregnancy rate in the 4XC-D5 group was significantly higher than that in the 4XC-D6 and 4CX-D6 groups (P’ < 0.001). Similarly, the clinical pregnancy rate in the 4CX-D5 group was significantly higher than that in the 4CX-D6 group and 4XC-D6 group (P’ < 0.001). In addition, the clinical pregnancy rate was significantly higher in the HQB-D6 group than in both the 4XC-D6 and 4CX-D6 groups (P’ < 0.001). Similarly, the clinical pregnancy rate was significantly higher in the HQB-D5 group than in either 4XC-D6 or 4CX-D6 groups (P’ < 0.001). No significant differences in pregnancy rates were found between the other subgroups. The LBR was significantly higher in both the 4XC-D5 and 4CX-D5 groups compared to the 4XC-D6 group (P’ < 0.001). Additionally, the HQB-D6 group had a significantly higher LBR compared to the 4XC-D6 group and 4CX-D6 group (P’ < 0.001). Furthermore, the LBR in the HQB-D5 group was significantly higher than both the 4XC-D6 and 4CX-D6 groups (P’ < 0.001). No statistically significant differences were found in LBR between the other subgroups. There was a significant difference in first trimester abortion rate among groups (P = 0.014). Subgroup analysis revealed that first trimester abortion rate in the HQB-D5 group was significantly higher than in the 4XC-D6 group (P’ < 0.001). No significant differences in first trimester abortion rate were found between any other subgroups.

Analysis results of subgroups showed that there was no difference in pregnancy and perinatal outcome between the delayed formation of D6 HQB and D5 blastocysts whether they were HQB or not (HQB-D5 vs. HQB-D6, HQB-D5 vs. 4XC-D5, HQB-D5 vs. 4CX-D5, HQB-Q6 vs. 4XC-D5, HQB-Q6 vs. 4CX-D5, P’ > 0.003). For LQB, the clinical pregnancy rate of D5 was higher than that of D6 (4XC-D5 vs. 4XC-D6, 4CX-D5 vs. 4CX-D6, P’ < 0.001), D5 was also better than D6 in LBR for those with ICM rating B or above (4XC-D5 vs. 4XC-D6, P’ < 0.001), while there was no difference between D5 and D6 for those with ICM rating C (4CX-D5 vs. 4CX-D6, P’ = 0.047) (**Supplementary 2**). For first trimester abortion rates, it is difficult to draw useful conclusions, and further research is needed.

### 3.3 Single factor and multivariate regression analysis

**Table 3** showed that single factor and multivariate regression analysis were performed to adjust for potential confounders: female age, infertility duration, female BMI, basal FSH level, basal AMH level, endometrial thickness, quality of blastocysts and the day of blastocyst vitrification. First, single factor regression analysis was conducted, and then variables with a P value of less than 0.1 were included in the multivariate regression analysis. The following variables were found to be independently associated with LBR. Female age was negatively correlated with LBR (OR = 0.95; 95% CI: 0.93–0.98; P < 0.001), LBR decreased by about 5.0% with each year of increase in female age. The day of blastocyst vitrification was independently associated with LBR (OR = 1.68; 95% CI: 1.31-2.14; P < 0.001), LBR of implanted on D5 blastocysts was about 1.68 times higher than that of blastocysts implanted on D6 blastocysts. Similarly, the quality of blastocysts was independently associated with LBR (OR = 1.57; 95% CI: 1.26-1.97; P < 0.001), LBR of HQB group was about 1.57 times higher than that of LQB group. After adjustment, AMH was no longer an influencing factor for LBR (P = 0.386). Infertility duration, female BMI, basal FSH and endometrial thickness were not significantly correlated with LBR (P > 0.05).

**Table 3:** Risk factors affecting the LBR after frozen-thawed blastocyst transfer, as shown by single factor and multivariate regression analysis.

Variables	OR (95% CI)	P	Adjusted OR (95% CI)	P
Female age	0.94 (0.92-0.96)	< 0.001	0.95 (0.93-0.98)	< 0.001
Infertility duration	0.96 (0.93-1.00)	0.065	0.99 (0.95-1.03)	0.595
Female BMI	0.99 (0.96-1.02)	0.654		
Basal FSH	1.01 (0.97-1.05)	0.617		
Basal AMH	1.04 (1.01-1.06)	0.001	1.01 (0.99-1.04)	0.386
Endometrial thickness	1.01 (0.95-1.07)	0.791		
D5 vs. D6				
D6	1.00		1.00	
D5	2.06 (1.64-2.59)	< 0.001	1.68 (1.31-2.14)	< 0.001
Quality of blastocysts				
LQB	1.00		1.00	
HQB	1.93 (1.57-2.37)	< 0.001	1.57 (1.26-1.97)	< 0.001

OR, odds ratio; 95% CI, 95% confidence interval; FSH, follicle-stimulating hormone; AMH, anti-mullerian hormone; HQB, high-quality blastocysts; LQB, low-quality blastocysts; D5, day 5; D6, day 6.

## 4 Discussion

With the popularization of vitrification and single blastocyst transfer, it is really important to select an embryo with higher potential for implantation. The effect of the delayed blastulation on the developmental potential of the embryo remains controversial. Our results show that in the frozen-thawed single blastocyst transfer cycles, D5 blastocysts regardless of HQB or LQB and D6 HQB at stage 4 have the same clinical outcome, suggesting that they have similar blastocyst development potential. It’s unlike anything we have seen before.

The difference in pregnancy outcomes among blastocyst groups may be related to serum AMH concentrations. Scheffer et al. and De Conto et al. showed that serum AMH concentration was related to embryo development and blastocyst formation, but too high or too low AMH concentration was not conducive to blastocyst formation (12, 13). In our study, AMH in HQB-D5 was significantly higher than in LQB groups regardless of D5 or D6, suggesting that it was related to the quality of the blastocysts formed. However, the serum AMH level did not emerge as an independent predictor of LBR in the multivariate regression analysis. Victoria M et al. argued that AMH appears to be a weak independent predictor of qualitative outcomes such as implantation, pregnancy, and live birth (14). Furthermore, this difference may be related to the growth rate of embryo. Hashimoto et al. reported a lower pregnancy rate in slow-growing embryos than in normally developing embryos. Studies have found that the incidence of abnormal spindles was high in the group of embryos showing slow development (15). Finally, this difference may be explained by the aneuploidy rate. D6 blastocysts, which showed slower development, had a higher aneuploidy rate than did D5 blastocysts, which showed faster development. Because PGT (Preimplantation genetic testing) was not done, relevant data on aneuploidy were not available. But Taylore et al. calculated that those embryos which did not blastulate until day 6 had a 10% increase in aneuploidy rates compared with those that blastulated on day 5. This difference may be the reason for the lower pregnancy outcome in D6 blastocysts (16).

Single factor and multivariate regression analysis were performed to determine the factors affecting LBR. We found that the age of the female patient was an independent factor affecting LBR and was also one of the most significant predictors of live birth in the frozen-thawed single blastocyst transfer cycles as PGT was not done. However, the effect of the blastocyst development rate on live birth needs to be determined on the basis of morphology. It appears that for high-quality blastocysts, the development rate has no effect on the LBR, but for the LQB with a low morphological score, the development rate has a significant effect. Our results were consistent with these literatures, with one exception, we found that low-quality D5 blastocysts and HQB blastocysts at stage 4 had the same developmental potential (8, 17).

However, Desai et al. reported there was no correlation between female age and clinical pregnancy (OR = 0.53; 95% CI: 0.15-1.77; P = 0.30) and LBR (OR = 0.31; 95% CI: 0.09-1.04; P = 0.06), and they observed that the day of blastocyst formation was strongly predictive of clinical outcome (18). An embryo blastulating by culture D5 was 3 times as likely as an embryo blastulating by culture D6 to give rise to a clinical pregnancy (OR = 3.08; 95% CI, 1.88-5.12; P < 0.001) as well as live birth (OR = 2.93; CI: 1.79-4.85; P < 0.001). Instead of considering blastocyst quality in their regression model, they included the expansion grade of the blastocyst. The chance of clinical pregnancy (OR = 4.05; 95% CI: 2.31-7.28; P < 0.001) and live birth (OR = 3.84; 95% CI: 2.15-7.09; P < 0.001) was about 4 times higher if an expanded blastocyst was transferred. Another study reported by Richter et al. showed that the probability of live birth per cycle declined significantly with age at cryopreservation (P < 0.0001) (19). Further study showed that there were no apparent differences in birth outcomes associated with expansion and transfer on D5 versus D6 after vitrification. Live birth per transfer (44.8% vs. 45.7%; P = 0.73) and live born children per embryo (36.9% vs. 36.4%; P = 0.85) were both nearly identical between D5 and D6 for vitrified blastocysts. Andrea Abdala et al. reported that in spite of the higher clinical outcomes with D5 blastocysts, clinical pregnancy rate with positive fetal heartbeat was more affected by endometrial thickness, patient age, BMI, and ICM grade C rather than biopsy day or endometrial preparation protocol (20). This difference between our finding and Desai et al.’s finding can be explained by the transfer of a high number of embryos to elderly patients in the latter’s study, which was associated with an increased risk of pregnancy failure due to aneuploidy. In addition, the blastocyst culture system and implantation strategy of different centers also affect clinical outcomes.

Our study is limited by its retrospective design and the unbalanced sample sizes in each group. Furthermore, it should be noted that the sample size of the HQB-D5 group was much larger than the other groups due to the preference for high-quality D5 blastocysts transfer. In addition, we performed an observational analysis of blastocysts at stage 4, and it is not known whether the same results are found for blastocysts at other stages. However, our study leads to a new conclusion, different from the previous literature, that D5 blastocysts, whether superior or not, have the same pregnancy and perinatal outcomes as D6 HQB. Our study also sheds new light on the definition of a good-quality blastocyst and provides a better understanding of the developmental potential of blastocysts.

## 5 Conclusion

Based on our findings that D6-expanded HQB has the same developmental potential as D5 expansion blastocysts, we recommend that when developing implantation strategies, we prioritize the selection of expanded HQB regardless of D5 and D6, with no difference in clinical outcomes. For LQB, D5-expanded blastocysts are preferred for transfer, but further randomized controlled studies with large samples are required.

## Data availability statement

The original contributions presented in the study are included in the article/**Supplementary Material**. Further inquiries can be directed to the corresponding authors.

## Ethics statement

All procedures performed in studies involving human participants were in accordance with the ethical standards of the institution and with the 1964 Declaration of Helsinki and its later amendments. The current study was approved by the Ethics Committee of the Ningbo Women and Children’s Hospital (ID: EC2022-M013). All patient privacy data in the study was desensitized, the need for informed consent was waived by the Ethics Committee of Ningbo Women and Children’s Hospital.

## Author contributions

JH: The conception and design of the study, writing-original draft preparation, funding acquisition. JZ: Conception and design of the study, acquisition and interpretation of data, funding acquisition. JL: Manuscript revision for important intellectual content. HS: Scientific and English revision of the manuscript. HW: Scientific and English revision of the manuscript and data curation. BZ: Scientific and English revision of the manuscript. KL: Acquisition of data and drafting the article. CR: Acquisition and interpretation of data. LZ: Funding acquisition, final approval of the version to be submitted.
