# Supplementary material for: D6 high-quality expanded blastocysts and D5 expanded blastocysts have similar pregnancy and perinatal outcomes following single frozen blastocyst transfer
Source: Front Endocrinol (Lausanne). 2023 Nov 9;14:1216910. doi: 10.3389/fendo.2023.1216910 (PMC10666767; doi:10.3389/fendo.2023.1216910)
Supplement: Supplementary file 1 [file Table_1.docx]

**Supplementary 1** Results of basal AMH level comparison between subgroups

| Comparison groups | Z | AMH |
| --- | --- | --- |
| HQB-D5 vs. HQB-D6 | -2.428 | 0.015 |
| 4XC-D5 vs. 4XC-D6 | -1.016 | 0.310 |
| 4CX-D5 vs. 4CX-D6 | -1.092 | 0.275 |
| HQB-D5 vs. 4XC-D5 | -3.220 | 0.001 |
| HQB-D5 vs. 4CX-D5 | -4.636 | < 0.001 |
| 4XC-D5 vs. 4CX-D5 | -0.574 | 0.566 |
| HQB-D6 vs. 4XC-D6 | -2.002 | 0.045 |
| HQB-D6 vs. 4CX-D6 | -2.463 | 0.014 |
| 4XC-D6 vs. 4CX-D6 | -0.352 | 0.725 |
| HQB-Q6 vs. 4XC-D5 | -0.848 | 0.396 |
| HQB-Q6 vs. 4CX-D5 | -1.578 | 0.114 |
| HQB-D5 vs. 4XC-D6 | -4.914 | < 0.001 |
| HQB-D5 vs. 4CX-D6 | -5.022 | < 0.001 |
| 4XC-D5 vs. 4CX-D6 | -1.511 | 0.131 |
| 4XC-D6 vs. 4CX-D5 | -0.664 | 0.507 |

HQB, high-quality blastocysts; AMH, anti-mullerian hormone; D5, day 5; D6, day 6. X stands for either A or B.
