# Supplementary material for: D6 high-quality expanded blastocysts and D5 expanded blastocysts have similar pregnancy and perinatal outcomes following single frozen blastocyst transfer
Source: Front Endocrinol (Lausanne). 2023 Nov 9;14:1216910. doi: 10.3389/fendo.2023.1216910 (PMC10666767; doi:10.3389/fendo.2023.1216910)
Supplement: Supplementary file 2 [file Table_2.docx]

**Supplementary 2** Results of clinical pregnancy, LBR and first trimester abortion rate comparison between subgroups

| Comparison groups | P’ | | |
| --- | --- | --- | --- |
|  | Clinical pregnancy | LBR | First trimester abortion rate |
| HQB-D5 vs. HQB-D6 | 0.575 | 0.371 | 0.359 |
| 4XC-D5 vs. 4XC-D6 | < 0.001 | < 0.001 | 0.045 |
| 4CX-D5 vs. 4CX-D6 | < 0.001 | 0.047 | 0.096 |
| HQB-D5 vs. 4XC-D5 | 0.141 | 0.102 | 0.453 |
| HQB-D5 vs. 4CX-D5 | 0.242 | 0.123 | 0.191 |
| 4XC-D5 vs. 4CX-D5 | 0.393 | 0.857 | 0.429 |
| HQB-D6 vs. 4XC-D6 | < 0.001 | < 0.001 | 0.041 |
| HQB-D6 vs. 4CX-D6 | < 0.001 | < 0.001 | 0.256 |
| 4XC-D6 vs. 4CX-D6 | 0.236 | 0.009 | 0.006 |
| HQB-Q6 vs. 4XC-D5 | 0.454 | 0.517 | 0.966 |
| HQB-Q6 vs. 4CX-D5 | 0.685 | 0.678 | 0.824 |
| HQB-D5 vs. 4XC-D6 | < 0.001 | < 0.001 | < 0.001 |
| HQB-D5 vs. 4CX-D6 | < 0.001 | < 0.001 | 0.472 |
| 4XC-D5 vs. 4CX-D6 | < 0.001 | 0.017 | 0.289 |
| 4XC-D6 vs. 4CX-D5 | < 0.001 | < 0.001 | 0.054 |

HQB, high-quality blastocysts; LBR, live birth rate; D5, day 5; D6, day 6. X stands for either A or B.
